# Lymphatic filariasis, infection status in *Culex quinquefasciatus* and *Anopheles* species after six rounds of mass drug administration in Masasi District, Tanzania

**DOI:** 10.1186/s40249-021-00808-5

**Published:** 2021-03-01

**Authors:** Eliza Lupenza, Dinah B. Gasarasi, Omary M. Minzi

**Affiliations:** 1 Department of Parasitology and Medical Entomology, School of Public Health and Social Sciences, Muhimbili University of Health and Allied Sciences, Box 65011, Dar es Salaam, Tanzania; 2 Department of Clinical Pharmacy, School of Pharmacy, Muhimbili University of Health and Allied Sciences, Box 65013, Dar es Salaam, Tanzania

**Keywords:** Lymphatic filariasis, *Wuchereria bancrofti*, *Culex quinquefasciatus*, *Anopheles gambiae*, *Anopheles funestus*, Mass drug administration, Infection rate

## Abstract

**Background:**

Lymphatic filariasis (LF) elimination program in Tanzania started in 2000 in response to the Global program for the elimination of LF by 2020. Evidence shows a persistent LF transmission despite more than a decade of mass drug administration (MDA). It is advocated that, regular monitoring should be conducted in endemic areas to evaluate the progress towards elimination and detect resurgence of the disease timely. This study was therefore designed to assess the status of *Wuchereria bancrofti* infection in *Culex quinqefasciatus* and *Anopheles* species after six rounds of MDA in Masasi District, South Eastern Tanzania.

**Methods:**

Mosquitoes were collected between June and July 2019 using Center for Diseases Control (CDC) light traps and gravid traps for indoor and outdoor respectively. The collected mosquitoes were morphologically identified into respective species. Dissections and PCR were carried out to detect *W. bancrofti* infection. Questionnaire survey and checklist were used to assess vector control interventions and household environment respectively. A Poisson regression model was run to determine the effects of household environment on filarial vector density.

**Results:**

Overall, 12 452 mosquitoes were collected of which 10 545 (84.7%) were filarial vectors. Of these, *Anopheles gambiae* complex*, An. funestus* group and *Cx. quinquefasciatus* accounted for 0.1%, 0.7% and 99.2% respectively*.* A total of 365 pools of *Cx. quinquefasciatus* (each with 20 mosquitoes) and 46 individual samples of *Anopheles* species were analyzed by PCR. For *Cx. quinquefasciatus* pools, 33 were positive for *W. bancrofti*, giving an infection rate of 0.5%, while the 46 samples of *Anopheles* species were all negative. All 1859 dissected mosquitoes analyzed by microscopy were also negative. Households with modern latrines had less mosquitoes than those with pit latrines [odds ratio (*OR*) = 0.407, *P* < 0.05]. Houses with unscreened windows had more mosquitoes as compared to those with screened windows (*OR* = 2.125, *P* < 0.05). More than 80% of the participants own bednets while 16.5% had no protection.

**Conclusions:**

LF low transmission is still ongoing in Masasi District after six rounds of MDA and vector control interventions. The findings also suggest that molecular tools may be essential for xenomonitoring LF transmission during elimination phase.

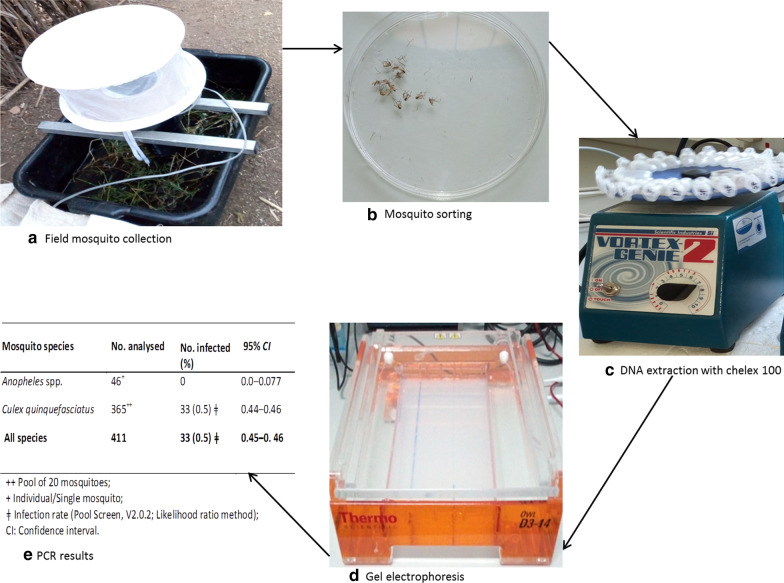

## Background

Lymphatic filariasis (LF) is one of the leading causes of disability worldwide, affecting more than 120 million people in 80 countries [1, 2]. In sub-Saharan Africa, LF is caused by the filarial nematode *Wuchereria bancrofti,* transmitted by mosquitoes of the species of *Culex quinquefasciatus*, *Anopheles gambiae* s.l. and *Anopheles funestus* [3–5].

LF has been targeted as a public health problem for elimination by the World Health Assembly due to the fact that, the disease causes disability and may be irreversible if not detected and treated on time. The World Health Organization (WHO) launched the Global Programme for Elimination of Lymphatic Filariasis in 2000 with the aim of interrupting and eventually halting the transmission through repeated mass drug administration (MDA) of anthelminthics. Depending on the country situation, ivermectin (IVM) or diethylcarbamazine citrate (DEC) in combination with albendazole (ALB) were recommended [6]. The effect of IVM and DEC is to kill microfilariae [7, 8], albendazole has no effect on LF microfilaria so the addition of ALB in MDA is meant to synergize the elimination of soil transmitted helminths (STH) [9]. DEC is not recommended in some countries of sub-Saharan Africa due to the co-endemicity with onchocerciasis. Administration of DEC in patients with onchocerciasis causes serious adverse effects such as encephalopathy, confusion, stupor, or coma [10]. Therefore, in many countries of sub-Saharan Africa, IVM and ALB are used for MDA campaigns against LF. In Tanzania, MDA involves an annual single dose administration of IVM plus ALB. After five rounds of annual MDA, the prevalence of microfilaria (MF) in endemic settings was expected to fall below 1% and hence reducing the potential for new transmission by mosquitoes [11]. The success of this strategy is evidenced in Western Pacific Region including Cambodia, Cook Islands, Egypt, Maldives, Marshall Islands, Niue, Sri Lanka, Thailand, Tonga, and Vanuatu where the MF prevalence fell below 1% after several rounds of implementing MDA with DEC and ALB [12–14]. Nevertheless, the elimination target has not been met in Sub-Saharan Africa after more than a decade of MDA, but there has been remarkable progress towards LF control and elimination in the region. Recently, Togo was declared as the first country in sub-Saharan Africa to achieve LF elimination following six rounds of MDA with ivermectin and albendazole using a network of community health workers [15].

In addition to MDA which is the main strategy for LF elimination programs, there has been a growing recognition on the potential role of vector control as a supplementary component to MDA [16–18]. Some studies demonstrated that, the use of insecticide treated bed nets (ITNs) resulted in reduction in prevalence and transmission of LF [19–21]. A study conducted in Papua New Guinea revealed a significant decrease in *W. bancrofti* infection rate among *Anopheles* mosquitoes from 1.8% to 0.4% following distribution of ITNs [19]. Promoting vector control strategies in addition to MDA may have a significant contribution towards elimination of LF.

LF is widespread in many regions of Tanzania with an estimated six million people with disabilities due to the disease [22]. In response to WHO efforts on LF elimination, the National Lymphatic Filariasis Elimination program (NLFEP) begun its operations in 2000, using ivermectin (150–200 μg/kg) and albendazole (400 mg) for individuals aged five years and above in selected endemic areas [22]. Since then, there have been evidence of decline in LF transmission in human populations [23–27]. Low infection and infectivity rates in mosquitoes have been demonstrated by studies in North Eastern Tanzania [26, 28] and Rufiji, South Eastern Tanzania [24].

Despite the reported decline in LF in Tanzania, the results from a recent entomological study established evidence of potential for on-going transmission of *W. bancrofti* in Mafia Island after 15 rounds of MDA [29]. It is therefore essential to conduct disease monitoring surveys both in areas undergoing MDA and after stopping MDA in order to evaluate the progress towards elimination and early detection of resurgence respectively. In addition to human blood testing for the presence of the parasites and detection of filarial antigenemia, xenomonitoring in filarial vectors has been considered as an integral component in monitoring the impact of MDA [16, 29, 30]. Xenomonitoring provides real time estimate of infection status where mosquitoes can be collected and assessed either through dissections to find the filarial larvae, or through the use of molecular methods to detect the DNA of the filarial worms [28, 29].

The implementation of MDA campaigns in Masasi district, South Eastern Tanzania, started in the year 2012, with a baseline circulating filarial antigen (CFA) prevalence of 11.7%. However, there is paucity of information on the current infection status in human and vector populations in the district. This study was therefore designed to assess the status of *Wuchereria bancrofti* infection in *Culex quinqefasciatus* and *Anopheles* species, after six rounds of MDA in Masasi District.

## Methods

### Study site

The study was conducted in Masasi District (10.7348° S, 38.8044° E) in Mtwara Region. It is bordered to the North by Lindi Region, to the East by the Newala District, to the South by the Ruvuma River and Mozambique, and to the West by Nanyumbu District. The population of the area is 247 993 [32], the inhabitants are mainly Makonde, Makua and Wayao tribes. The average annual temperature of the area is 25.4 ℃ and average annual rainfall is 1024 mm with humidity of 82%. The main socio-economic activities include cashewnut farming. Masasi is an endemic district for LF; it is among the districts which are currently under MDA with ivermectin and albendazole. The implementation of LF elimination activities in the district started in 2012, with baseline CFA prevalence of 11.7% which had significantly declined to 4.7% in 2018 following six rounds of MDA.

### Study design

**Fig. 1:**
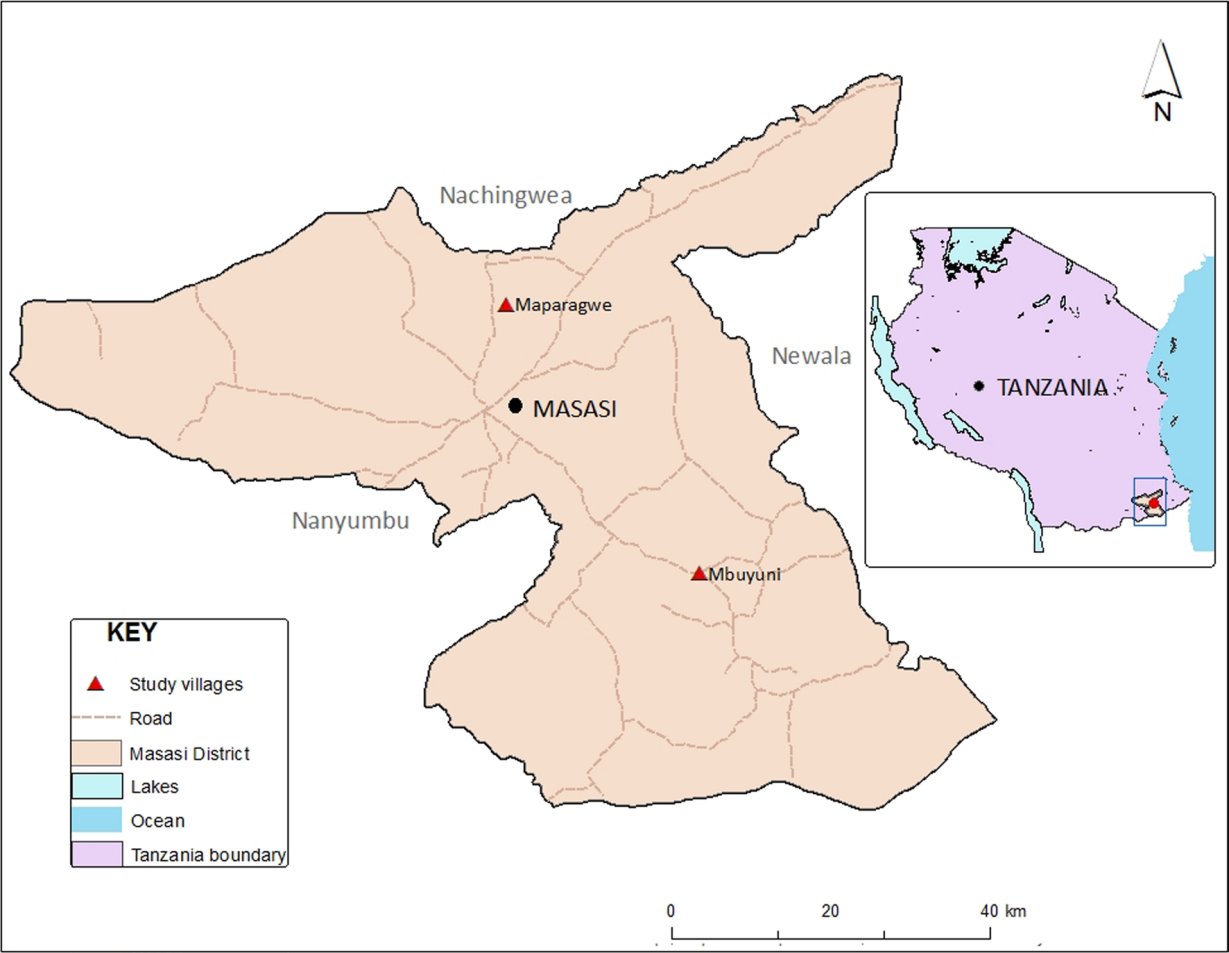
A map showing the location of the two study villages, Maparagwe and Mbuyuni within Masasi District, Southeastern Tanzania

This was a cross sectional study conducted in two villages namely Maparagwe and Mbuyuni which were purposely selected from 20 villages of Masasi District Council because they were considered to have high transmission. Maparagwe is in the North, 10 km from the town center, while Mbuyuni is in the South, approximately 40 km from Masasi town center (Fig. 1). For each of the two villages, 25 households were selected giving a total of 50 households. Maparagwe Village consists of five hamlets, in each hamlet, five households were randomly selected. While Mbuyuni consists of six hamlets, four households were randomly selected from each of the hamlets. The remaining one house was selected from the randomly selected hamlet in the list of the six hamlets taking into consideration the distance from the previously selected households. Prior to commencing the study; meetings were conducted with village leadership, community drug distributors (CDDs) and influential people in each respective village. The purpose of the study and methods were clarified to the community representatives. Each of the selected households was visited by the research team to seek for informed consent from the head of the household regarding participation in the study. The environmental features of the household including type of house, latrine type, presence of stagnant waters, tall grasses/bushes were recorded. A survey was also conducted to collect information on bed net ownership and use, indoor residual sprays (IRS), and mosquito repellent use as well as coverage of ITNs in the community.

### Mosquito collection procedures

Mosquito collection was conducted from mid-June 2019 to late July 2019. Two types of traps were used in mosquito trapping, the U.S. Centers for Disease Control and Prevention (CDC) Light traps (John W. Hock Co. Gainesville, FL) were set indoor, and CDC Gravid traps (John W. Hock Co. Gainesville, FL), containing grass infusion were set in the outdoor position. Light traps are effective in collecting *Anopheles* mosquitoes while gravid traps are effective in collecting *Cx. quinquefasciatus* [28, 33]. The presence of grass infusion provide oviposition cues and potentially collect gravid mosquitoes as they approach the organic infusion in the pan below the trap.

Each night, five light traps were set indoors, and five gravid traps were set outdoors at the selected houses. Two traps were set at each house, the indoor and outdoor trap were only set twice throughout the time of the study. Indoor mosquito collection was conducted in one room with an occupant(s) sleeping under mosquito nets. Light traps were set near occupied bed nets at the foot end, at approximately 1.5 m from the ground, as described previously [34, 35]. Gravid traps containing 4 L of grass infusion were placed outdoor as described in previous studies [28]. Briefly, grass infusion was prepared by soaking grass in water for 24 h in a covered plastic bucket to prevent any mosquito oviposition. Both light and gravid traps were set between 18:00 and 19:00 PM and collected early in the morning of the next day between 6:00 and 7:00 AM. Trap nets were removed from the traps and returned to the processing area for morphological identification, dissection and packaging.

### Morphological identification and packaging

The collected mosquitoes were morphologically identified to species, based on available keys for *Anophelines* [36] and *Culicines* [37]. Briefly, mosquitoes were identified to their respective species based on common structural features including: wings, abdomen, head, thorax, legs, mouth parts and scales. After identification, mosquitoes were counted and segregated into filarial and non-filarial vectors. Individual filarial vectors were recorded in a mosquito recording sheet. Female *Cx. quinquefsciatus* mosquitoes were stored in dry clean eppendorf tubes with silica gel in a pool of 20 mosquitoes while *Anopheles* species were stored individually for molecular analysis by PCR.

### Detection of *Wuchereria bancrofti* in mosquitoes

#### Dissection and detection of *W. bancrofti* larval stages by microscopy

A total of 1822 freshly killed female *Cx. quinquefasciatus* and 37 *Anophele*s species were dissected to assess if they harbored any stage of *W. bancrofti* larvae. Dissection standard procedures were followed, as previously described [29, 38]. Dissection results were recorded into a designed sheet. Later on, the data was entered into Microsoft excel data-base then imported into SPSS version 22 (SPSS, Inc., IL, USA) for analysis. Mosquito infection was defined as the presence of any larval stages, first stage larvae (L1), second stage larvae (L2), and/or third stage larvae (L3), while mosquito infectivity was defined as the presence of L3 larvae in any of the body segments [39].

### Detection of *W*. *bancrofti* by polymerase chain reaction (PCR)

#### Deoxyribonucleic acid (DNA) extraction from mosquitoes

Mosquito genomic DNA (gDNA) extraction was carried out by using the Chelex-100 Resin with modifications as described earlier [3]. Briefly, the pulsating vortex machine was used for homogenizing mosquitoes (20 in each tube) in 250 μl of 10% chelex buffer solution (C7901, Sigma, CA, USA). The Extracted gDNA was analyzed for the presence of *W. bancrofti* DNA by PCR targeting highly repetitive amino acid sequences of *W. bancrofti* DNA. Each reaction mixture consisted of 0.125 μmol/L of forward and reverse primers, 10 μl Hot StartTaqTEMPase polymerase master mix and 2 μl of DNA extract. The amplified DNA for *W. bancrofti* specimen were separated based on their fragment size by gel electrophoresis and visualized under ultra violet light as previously described [40].

### Assessment of household characteristics and surrounding environment

A survey was conducted to collected information on the characteristics of households and the surrounding environment. This information included type of the house based on construction materials which were classified as, brick with iron roof, brick with grass roof and mud with grass roof. Window screening, the type of latrine, the presence of tall grasses and bushes around the house, and the presence of stagnant waters was also recorded (Additional file 1).

### Assessment of vector control strategies in the study community

Pre-tested questionnaires were administered to 588 individuals in the study area to obtain information on use of vector control interventions. The questions included but were not limited to: whether they own and use bed net(s), whether the bed nets were insecticide treated or not treated. Information on other vector control interventions was also sought, such as use of IRS and mosquito repellent (Additional file 2).

### Data analysis

All data were entered in Excel spreadsheets (Microsoft corp., Redmond, USA) and transferred to SPSS version 20.0 (SPSS, Inc., IL, and USA). The ‘infection rate’ of the dissected mosquitoes was calculated as the percentage of mosquitoes infected with any stages of *W. bancrofti* that is L1, L2 or L3. While the ‘infectivity rate’ was calculated as the percentage of mosquitoes infected with infective larvae (L3) as previously described [29]. For pooled mosquitoes which were analyzed by PCR technique; The Pool Screen (v.2.02) software (Department of Biostatistics and Division of Geographic Medicine, University of Alabama at Birmingham, USA) [22] was used to calculate the probability that any one of the mosquitoes is infected with any stage of *W. bancrofti*. Two sample *t*-tests for proportions were used to compare the infection rates among mosquitoes caught indoor and outdoor, and between the two villages. A *P*-value of less than or equal to 0.05 was considered statistically significant. Poisson regression model was run to assess the influence of household environments on vector density. Whereby, vector density was modeled as the function of house type, latrine type, and window screen, presence of tall grasses, bushes and stagnant waters around the house. The differences in bed net ownership between the two villages were compared using chi-square tests, where *P* ≤ 0.05 were considered as statistically significant.

## Results

### Mosquito populations and composition

A total of 12 452 mosquitoes were collected in the two villages during the study period, whereby, 7860 (63.1%) were collected from Maparagwe and 4592 (36.9%) from Mbuyuni village. Of the total mosquitoes collected, 1868 (15%) were collected indoor, while 10 583 (85%) were collected outdoor. Majority 10 545 (84.7%) of the collected mosquitoes were filarial vectors. The remaining 1907 (15.3%) were mosquitoes belonging to the species of *Culex sinilius*, *Coquilettidia* spp. and *Aedes* spp. The composition of the filarial vectors included *An. gambiae* complex 15 (0.1%), *An. funestus* group 73 (0.7%) and *Cx. quinquefasciatus* 10 457 (99.2%) (Table 1).

**Table 1:** Mosquito populations and composition from two villages in Masasi District

Mosquito taxa	Maparagwe Village		Mbuyuni Village		Total collection, *n* (%)
	Indoor, *n* (%)	Outdoor, *n* (%)	Indoor, *n* (%)	Outdoor, *n* (%)	
*Anopheles gambiae* complex	15 (1.1)	0 (0.0)	0 (0.0)	0 (0.0)	15 (0.1)
*An. funestus* group	63 (4.4)	0 (0.0)	10 (2.2)	0 (0.0)	73 (0.6)
*Culex quinquefasciatus*	1199 (84.3)	5484 (85.2)	347 (77.6)	3427 (82.7)	10 457 (84)
Other mosquitoes	145 (10.2)	954 (14.8)	90 (20.1)	718 (17.3)	1907 (15.3)
Total by trap/village	1422	6438	447	4145	12 452

Other mosquito species include *Culex sinilius, Coquilettidia* spp. and *Aedes* spp.

### Microscopy examination

A total of 1822 female *Cx. quiquefasciatus* and 37 female *Anopheles* species from both CDC light and gravid traps were dissected and examined for infection with *W. bancrofti* larvae giving a total of 1859 dissected mosquitoes. None of the 1859 dissected mosquitoes were found to be infected with any of the larval stages (L1, L2 and /or L3) of *W. bancrofti* (Table 2).

**Table 2:** Vector infection rate by methods of analysis and study site

Analysis method	Mosquito species	No. analysed	No. infected (%)	95% *CI*	*P-*value
Microscope	*Anopheles* spp.	37	0	0.0–0.095	
	*Culex quinquefaciatus*	1822	0	0.0–0.001	
	All species	1859	0	0.0–0.001	
PCR	*Anopheles* spp.	46^+^	0	0.0–0.077	
	*Culex quinquefasciatus*	365^++^	33 (0.5) ǂ	0.44–0.46	0.1
	All species	411	33 (0.5) ǂ	0.45–0. 46	
Study site	Maparagwe	280	15 (0.3) ǂ	0.256–0.36	
	Mbuyuni	131	18(0.7) ǂ	0.61–0.77	0.004*
	All villages	411	33 (0.5) ǂ	0.45–0. 46	

*CI* confidence interval

^+ + ^Pool of 20 mosquitoes; ^+ ^Individual/Single mosquito; ǂ Infection rate (Pool Screen, V2.0.2; Likelihood ratio method); * Two sample test for proportion to compare mosquito infection rates between villages

### Molecular analysis of mosquito samples

Using PCR technique, a total of 365 pools of *Cx. quinquefasciatus* each containing 20 mosquitoes and 46 individual *Anopheles* species (*An. gambiae* and *An. funestus*) were tested for infection with *W. bancrofti*. Of the 365 pools of *Cx. quinquefasciatus,* 33 were found to be infected with *W.bancrofti*. All 46 *Anopheles* samples were found to be negative. Analysis by study site indicates a significant difference in infection rate between the two study villages (two sample, *t*-test for proportions, *P* = 0.004). For both species and study villages, the probability that any one mosquito in the pool was infected with any stage of *W. bancrofti* parasite was estimated at 0.5% (Table 2).

### Characteristics and environmental features of the sampled households

Overall, 94.0% (*n* = 47) of sampled households (*n* = 50) had pit latrines. Only, 14.0% (*n* = 7) of households had screened windows to prevent mosquito entry. None of them had stagnant waters around; this is because the survey was done during the dry season, between June and July. In addition to that the majority of the households 98% (*n* = 48) had no tall grasses and bushes around (Table 3).

**Table 3:** Characteristics and environmental features of the samples households

Features	Status/observation	Number of households *n* = 50
*House type*		
Bricks/grass roof	Yes	16
	No	34
Bricks/iron roof	Yes	32
	No	18
Mud/grass roof	Yes	2
	No	48
Window	Not screened	43
	Screened	7
*Latrine type*		
Modern	Yes	3
	No	47
Pit latrine	Yes	47
	No	3
Uses bednets	Yes	48
	No	2
Uses bednets + IRS	Yes	2
	No	48
Presence of tall grasses	Yes	2
	No	48
Presence of bushes around house	Yes	0
	No	50
Presence of stagnant water	Yes	0
	No	50

*IRS*  indoor residual sprays

### Effects of household characteristics and the surrounding environment on vector density

The results showed that, houses constructed with bricks had significantly fewer mosquitoes compared to those constructed with mud regardless of the roof type (iron sheets or grass) (*OR* = 0.638 and *OR* = 0.412, respectively *P* < 0.05). Houses with unscreened windows had two times more mosquitoes as compared to those with screened windows, (*OR* = 2.125, *P* < 0.05). Households with modern latrines had 60% times less mosquitoes as compared to the households with pit latrines (*OR* = 0.407, *P* < 0.05). Households without tall grasses around were four times more likely to have mosquitoes compared to the households with tall grasses in the surrounding (*OR* = 4.320, *P* < 0.05) (Table 4).

**Table 4:** Effects of household characteristics and surrounding environment on vector density (*n* = 50)

Variable	Category	*OR*	95% *CI*	*P*-value
House type	Bricks/grass roof	0.412	0.385–0.440	0.000
	Bricks/iron roof	0.644	0.606–0.684	0.000
	Mud/wood*	1		
Window	Not screened	2.125	1.970–2.293	0.000
	Screened*	1		
Type of latrine	Modern latrine	0.407	0.362–0.457	0.000
	Pit latrine*	1		
Presence of tall grasses around the house	No	4.320	3.461–5.391	0.000
	Yes*	1		

*P* ≤ 0.05 is significant

*OR* odds ratio, *CI* confidence interval

*Reference category

### Vector control interventions in the study community

**Fig. 2:**
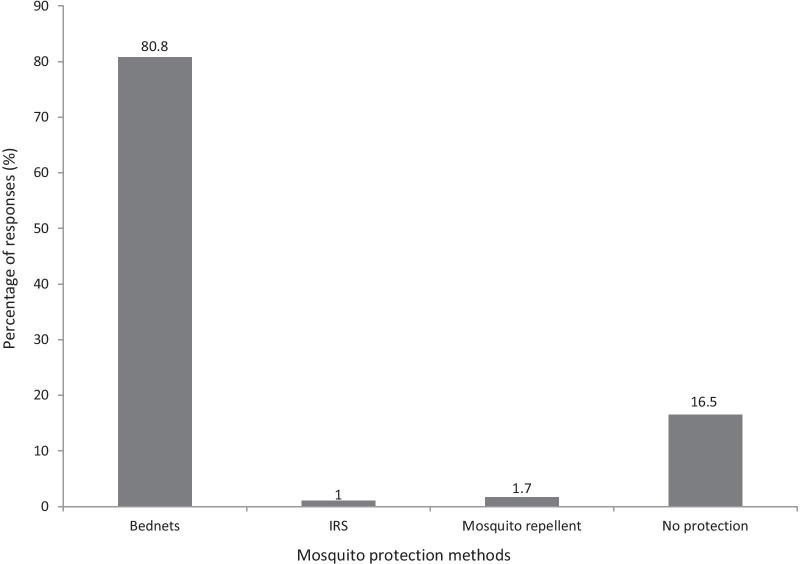
Vector control interventions in the study community, percentage (%) of respondents using bednet, indoor residual sprays (IRS), mosquito repellents, and those with no mosquito protection (*n* = 588)

**Fig. 3:**
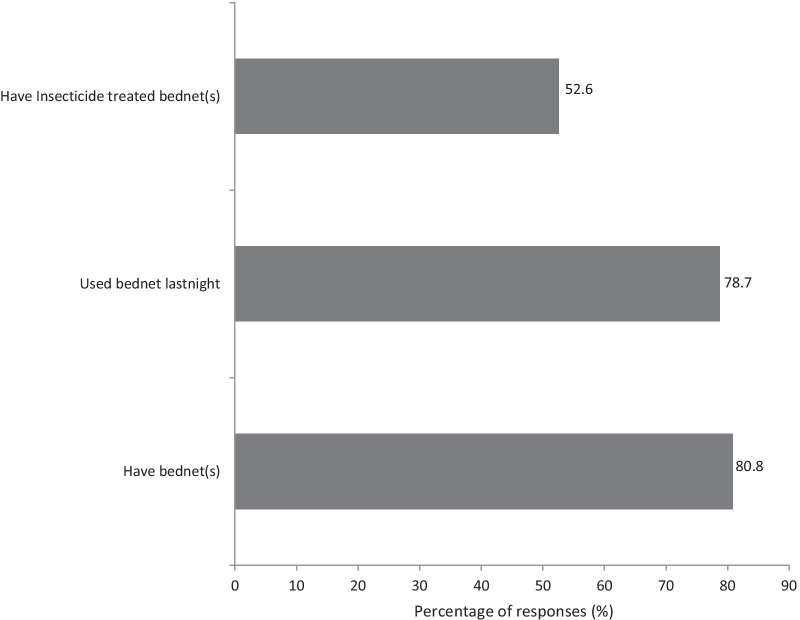
Percentage of responses on bednet ownership, use and coverage of insecticide treated bed nets among the study participants (*n* = 588)

Bednet use was the main vector control intervention where 80.8% (475/588) bednet ownership was recorded in the community. While only about two percent used mosquito repellent and IRS use only one percent (Fig. 2). The majority, 78.7% reported to have slept under bednets during the last night before the day of the interview and among those who own bednets; only 52.6% had ITNs (Fig. 3). Analysis of bednet ownership by village revealed no statistically significant differences between the two villages (Chi-square = 0.673, df = 1, *P* = 0.412).

## Discussion

The global programme for elimination of LF advocates annual MDA with ALB and IVM or DEC in specific endemic areas, complimented with regular monitoring of the disease status towards elimination. This study was therefore designed to assess the LF infection status in filarial vectors after six rounds of MDA in Masasi District.

The current study reports an infection rate of 0.5% in mosquitoes after six rounds of MDA which indicates a low ongoing LF transmission in the district. The obtained infection rate is above the cut-off point of 0.25%, a threshold that has been suggested for areas where *Culex* mosquitoes are the vectors [41]. This suggests a potential for persistent transmission which may be facilitated by presence of the parasite reservoirs [42]. In Tanga Region, where LF is also endemic, six rounds of MDA resulted into a decline in vector infection rate by 99.3% [43] and a follow up study reported no infection in mosquitoes after eight rounds of MDA in the same region [23]. It is therefore evident that, there is significant progress towards interrupting LF transmission in Tanzania using MDA interventions.

The infection rate obtained in this district is four times higher than the previously reported infection rate of 0.1% in Rufiji district after twelve rounds of MDA [24], which is in the same geographical region. But it is four times less than the infection rate of 1.7% reported in Mafia Island after 15 rounds of MDA [29]. The observed differences might be attributable to a number of factors, including but not limited to: the initial level of LF prevalence and density of microfilaremia, the competence and vectorial capacity of the local vectors as well as population coverage and compliance to MDA [42]. The effect of some of these factors is exemplified by the reports in Mafia Island and Masasi District, where the baseline CFA prevalence in Mafia Island was 49%, while that of Masasi District was 11.7%. The observed differences in the levels of infection rates were 1.7% vs 0.5% respectively; which may be inherent in the significantly different levels of infection status at the initiation of the MDA program. In addition the number of MDA rounds, the coverage and community compliance to the MDA programme would to a great extent influence the decline in parasite prevalence, microfilaria density and hence the transmission dynamics and vector infectivity levels [42].

The findings of this study indicate that, out of 1859 microscopically dissected mosquito samples, no single mosquito was found to be infected with any larval stages of *W. bancrofti*, while analysis by PCR revealed an infection rate of 0.5% (Table 2). These observations emphasize the superiority of molecular techniques for xenomonitoring in settings with low LF transmission [30, 31]. The findings of this study are corroborated by the findings of a similar study in Mafia Island, Tanzania which reported infection rates of 0.3% by microscopy technique vs 1.7% by PCR technique [29]. Given the existing low transmission levels in most endemic communities during the implementation of the elimination program, molecular based techniques may be an effective tool for xenomonitoring.

The current findings also indicate that, *Cx. quinquefasciatus* is a dominant vector of *W. bancrofti* in Masasi District, which accounted for 99.2% of the filarial vectors sampled. More mosquitoes were caught outdoor using CDC gravid traps compared to the indoor with CDC light traps. It is hypothesized that, the difference in trap performance may be due to the organic grass infusion attractant added to the CDC gravid traps.

The dominance of *Cx. quinquefasciatus* has been reported in a number of earlier studies [29, 44, 45] and has been considered as a dominant vector in urban areas [46]. Interestingly, in recent years studies have shown that *Cx. quinqufasciatus* is now a dominant vector both in rural and urban settings [47]. A study in North Eastern Tanzania, demonstrated a major shift in vector species composition; from predominantly *Anophelines* in the pre-MDA period to almost exclusively *Culicines* after six rounds of MDA [43]. This shift in vector composition has been linked to multiple factors including; environmental and climate changes as well as documented evidence that IVM may have effects on *Anopheles* species.

The current study demonstrated low population densities of *An. gambiae* and *An. funestus.* It is possible that the observed low densities of *Anopheles* species from this study may be linked to the effects of ivermectin which is administered annually in the study area. Increased mortality and decreased fecundity of *Anopheles* species taking blood meals on ivermectin treated individuals has been demonstrated in a number of clinical trials [48, 49], field reports [50, 51] and laboratory studies [52–54].

The current study findings also indicate that, some household characteristics including pit latrine and unscreened windows have influence on density of filarial vectors. Households with pit latrines had significantly higher counts of mosquitoes collected compared to households with modern latrines (Table 4). This observation is not surprising because pit latrines are known to be breeding habitats for *Culex* mosquitoes as they prefer to breed in polluted waters [45]. This view is corroborated by findings from an intervention study in which pit latrines and septic tanks, were treated with polystyrene beads in Dar es salaam region and the outcome was a significant reduction in densities of adult *Cx. quinquefasciatus* [55]. Unscreened windows were also associated with increased indoor mosquito densities which present increased biting rates to the house occupants and hence increased risk of LF infection. These findings coupled with the fact that *Cx. quinquefasciatus* seems to expand its horizons to both urban and rural areas; may suggest that, vector control focusing on environmental improvement may be an important factor in the LF elimination program in some of the endemic areas.

The National Malaria Control Programme in Tanzania has significantly contributed towards vector control, through its campaigns on mosquito net distribution to vulnerable groups (pregnant women and children). Thus high coverage of bednets in both villages was recorded in this study. The use of ITNs has been linked to the decline in populations of *Anopheles* species [56, 57]. Very low densities of *Anopheles* mosquitoes were observed in this study, which could be linked to both the use of ITNs and the IVM effects on these mosquito populations. Although nearly half of the bednet owners used un-treated bednets, it offers physical protection by preventing human-mosquito contact and hence reducing the risk of infection [58]. The use of ITNs may therefore be of value to both malaria and LF control programs and should be advocated by both programs, even though vector control is not advocated in the LF elimination strategy.

The lethal effects of IVM on *Anopheles* mosquitoes have been demonstrated [48, 50–52, 54, 59]. However, IVM does not seem to have lethal effects on *Culex* species [53]. Laboratory studies using adult *Cx. quinquefasciatus* fed on blood meals from volunteers treated with ivermectin revealed no effects on its survival, egg laying capacity and development of larvae [53]. Though, mortality of *Cx. quinquefasciatus* was reported when fed on chicken treated with 2000 µg/kg of ivermectin (about ten times the therapeutic dosage) [60]. In addition, *Cx. quinquefasciatus* are relatively tolerant to insecticides used for ITNs and IRS interventions [61, 62]. It may therefore be important to develop effective vector control tools against this mosquito species in order to maximize the impact of MDA.

The main limitation of the findings of this study is that, mosquito collection covered only 50 households, which may not be sufficient to represent the vector dynamics and infection status in the district. Furthermore, data collection was done during the dry season between June and July, which may have impacted the density of *Anopheles* mosquitoes. Earlier studies have reported that there is seasonal variation in the density of *Anopheles* mosquitoes with a decline during the dry season due to shortage of breeding habitats [63. Therefore, a longitudinal entomological survey is needed to assess, effects of seasonality on vector density, vector species, transmission dynamics and the potential risk factors.

## Conclusions

LF low transmission is still ongoing in Masasi District after six rounds of MDA and vector control interventions which are in place. There also seem to be a shift in filarial vector transmission and population dynamics in the rural setting with *Cx. quinquefasciatus* dominating over the *Anopheles* species. The findings also suggest that molecular tools may be essential for xenomonitoring in assessment of LF transmission during the elimination phase. Based on the findings of this study, it may be reasonable to suggest that, in order to halt LF transmission as per global LF elimination initiative, an integrated strategy is essential. Integrated vector control strategies including use of ITNs, environmental improvement and modernization of latrines to limit vector breeding habitats for *Cx. quinquefasciatus* may be an important addition to the MDA strategy.

## Supplementary Information

**Additional file 1:** Checklist on characteristics of the household and the surrounding environment.

**Additional file 2:** Questionnaires for community lymphatic filariasis screening in Masasi District council.

## Data Availability

The datasets used and/or analyzed during the current study are available from the corresponding author on reasonable request.

## Abbreviations

ALB: Albendazole
*CI*: Confidence interval
CDC: Center for disease control
CDDs: Community drug distributors
CFA: Circulating Filarial Antigen
DEC: Diethylcarbamizine
df: Degree of freedom
ITNs: Insecticide treated nets
IRS: Indoor residual spray
IVM: Ivermectin
LF: Lymphatic filariasis
LT: Light trap
MDA: Mass drug administration
*OR*: Odd ratio
PCR: Polymerase chain reaction
SPSS: Statistical package for Social Sciences

## References

[1] Hotez PJ, Kamath A (2009). Neglected tropical diseases in sub-Saharan Africa: review of their prevalence, distribution, and disease burden. PLoS Negl Trop Dis..

[2] Zeldenryk LM, Gray M, Speare R, Gordon SMW (2011). The emerging story of disability associated with lymphatic filariasis: a critical review. PLoS Negl Trop Dis.

[3] Rwegoshora RT, Pedersen EM, Mukoko DA, Meyrowitsch DW, Masese N, Malecela-Lazaro MN (2005). Bancroftian filariasis: patterns of vector abundance and transmission in two East African communities with different levels of endemicity. Ann Trop Med Parasitol.

[4] Yokoly FN, Zahouli JBZ, Méite A, Opoku M, Kouassi BL, de Souza DK (2020). Low transmission of *Wuchereria bancrofti* in cross-border districts of Côte d'Ivoire: a great step towards lymphatic filariasis elimination in West Africa. PLoS ONE.

[5] de Souza DK, Koudou B, Kelly-Hope LA, Wilson MD, Bockarie MJ, Boakye DA (2012). Diversity and transmission competence in lymphatic filariasis vectors in West Africa, and the implications for accelerated elimination of *Anopheles*-transmitted filariasis. Parasit Vectors.

[6] World Health Organization (2018). Global programme to eliminate lymphatic filariasis: progress report, 2017. Wkly Epidemiol Rec..

[7] Centers for Disease Control and Prevention. Lymphatic filariasis; guidance for evaluation and treatment. https://www.cdc.gov/parasites/lymphaticfilariasis/health professionals. Accessed 25 Sept 2020.

[8] Stolk WA, Oortmarssen GJ, Pani SP, De Vlas SJ, Subramanian S (2005). Effects of ivermectin and diethylcarbamazine on microfilariae and overall microfilaria production in bancroftian filariasis. Am J Trop Med Hyg..

[9] Ottesen EA, Ismail MM, Horton J (1999). The role of albendazole in programmes to eliminate lymphatic filariasis. Parasitol Today.

[10] Gardon J, Gardon-Wendel N, Kamgno J, Chippaux JP, Boussinesq M (1997). Serious reactions after mass treatment of onchocerciasis with ivermectin in an area endemic for* Loa loa* infection. Lancet..

[11] WHO/Department of Communicable Disease Prevention, Control and Eradication. Global programme to eliminate lymphatic filariasis: annual report on lymphatic filariasis 2001. Geneva. 2002;76.

[12] World Health Organization. Lymphatic filariasis, 2019. Fact sheet. https://www.who.int/news-room/fact-sheets/detail/lymphatic-filariasis. Accessed 2 Mar 2020

[13] Allen T, Taleo F, Graves PM, Wood P, Taleo G, Baker MC (2017). Impact of the Lymphatic Filariasis Control Program towards elimination of filariasis in Vanuatu, 1997–2006. Trop Med Health.

[14] Huppatz C, Capuano C, Palmer K, Kelly PM, Durrheim DN (2009). Lessons from the Pacific programme to eliminate lymphatic filariasis: a case study of 5 countries. BMC Infect Dis.

[15] Dorkenoo MA, Bronzan R, Yehadji D, Tchalim M, Yakpa K, Etassoli S (2018). Surveillance for lymphatic filariasis after stopping mass drug administration in endemic districts of Togo, 2010–2015. Parasit Vectors.

[16] World Health Organization. Defining the roles of vector control and xenomonitoring in the global programme to eliminate lymphatic filariasis. Report of the Informal Consultation. Geneva, 2002.

[17] Bockarie MJ, Pedersen EM, White GB (2009). Role of vector control in the global program to eliminate lymphatic filariasis. Annu Rev Entomol.

[18] Progress report 2000–2009 and strategic plan 2010–2020 of the global programme to eliminate lymphatic filariasis: halfway towards eliminating lymphatic filariasis. 2010. WHO. Geneva. https://apps.who.int/iris/handle/10665/44473.

[19] Reimer LJ, Thomsen EK, Tisch DJ, Henry-Halldin CN, Zimmerman PA, Baea ME (2013). Insecticidal bed nets and filariasis transmission in Papua New Guinea. N Engl J Med.

[20] Friedrich MJ (2013). Insecticidal bed nets help reduce lymphatic filariasis transmission. JAMA.

[21] Bøgh C, Pedersen EM, Mukoko DA (1998). Permethrin-impregnated bednet effects on resting and feeding behaviour of lymphatic filariasis vector mosquitoes in Kenya. Med Vet Entomol..

[22] Katholi CR, Toé L, Merriweather A (1995). Determining the prevalence of *Onchocerca volvulus* infection in vector populations by polymerase chain reaction screening of pools of black flies. J Infect Dis.

[23] Simonsen PE, Derua YA, Magesa SM, Pedersen EM, Stensgaard AS, Malecela MN (2014). Lymphatic filariasis control in Tanga Region, Tanzania: status after eight rounds of mass drug administration. Parasit Vectors.

[24] Jones C, Ngasala B, Derua YA, Tarimo D, Reimer L, Bockarie M (2018). Lymphatic filariasis transmission in Rufiji District, southeastern Tanzania: infection status of the human population and mosquito vectors after twelve rounds of mass drug administration. Parasit Vectors.

[25] Simonsen PE, Magesa SM, Dunyo SK, Malecela-Lazaro MN, Michael E (2004). The effect of single dose ivermectin alone or in combination with albendazole on *Wuchereria bancrofti* infection in primary school children in Tanzania. Trans R Soc Trop Med Hyg.

[26] Simonsen PE, Derua YA, Kisinza WN, Magesa SM, Malecela MN, Pedersen EM (2013). Lymphatic filariasis control in Tanzania: effect of six rounds of mass drug administration with ivermectin and albendazole on infection and transmission. BMC Infect Dis.

[27] Mshana HJ, Baraka V, Misinzo G, Makunde WH (2016). Current epidemiological assessment of bancroftian filariasis in Tanga region, northeastern Tanzania. J Trop Med.

[28] Irish SR, Stevens WMB, Derua YA, Walker T, Cameron MM (2015). Comparison of methods for xenomonitoring in vectors of lymphatic filariasis in northeastern Tanzania. Am J Trop Med Hyg.

[29] Derua YA, Rumisha SF, Batengana BM, Max DA, Stanley G, Kisinza WN (2017). Lymphatic filariasis transmission on Mafia Islands, Tanzania: evidence from xenomonitoring in mosquito vectors. PLoS Negl Trop Dis.

[30] Kouassi BL, De SDK, Goepogui A, Narh CA, King SA, Mamadou BS (2015). Assessing the presence of *Wuchereria bancrofti* in vector and human populations from urban communities in Conakry, Guinea. Parasit Vectors.

[31] Farid HA, Morsy ZS, Helmy H, Ramzy RM, El Setouhy MWG (2007). A critical appraisal of molecular xenomonitoring as a tool for assessing progress toward elimination of lymphatic filariasis. Am J Trop Med Hyg.

[32] Masasi District profile. In: Wikipedia. 2020. https://en.wikipedia.org/wiki/Masasi_District. Accessed 10 Oct 2020.

[33] Irish SR, Moore SJ, Derua YA, Bruce J, Cameron MM (2013). Evaluation of gravid traps for the collection of *Culex quinquefasciatus*, a vector of lymphatic filariasis in Tanzania. Trans R Soc Trop Med Hyg.

[34] Lines JD, Curtis CF, Wilkes TJ, Njunwa KJ (1991). Monitoring human-biting mosquitoes (Diptera: Culicidae) in Tanzania with light-traps hung beside mosquito nets. Bull Entomol Res.

[35] Mboera LEG, Kihonda J, Braks MAH, Knols BGJ (1998). Short report: Influence of centers for disease control light trap position, relative to a human-baited bed net, on catches of *Anopheles gambiae* and *Culex quinquefasciatus* in Tanzania. Am J Trop Med Hyg.

[36] Stolk WA, Van Oortmarssen GJ, Pani SP, De Vlas SJ, Subramanian S, Das PK (2005). Effects of ivermectin and diethylcarbamazine on microfilariae and overall microfilaria production in bancroftian filariasis. Am J Trop Med Hyg.

[37] Wallace AR, Wallace AR. Mosquitoes of the Ethiopian Region III. Culicine adults and pupae. London; 2012. p. 251–313.

[38] Goodman DS, Orelus JN, Roberts JM, Lammie PJ, Streit TG (2003). PCR and mosquito dissection as tools to monitor filarial infection levels following mass treatment. Filaria J.

[39] World Health Organization (2013). Lymphatic filariasis: a handbook of practical entomology for national lymphatic filariasis elimination programs.

[40] Vythilingam I, Boaz L (1998). Detection of *Brugia malayi* in mosquitoes by the polymerase chain reaction. J Am Mosq Control Assoc.

[41] Farid HA, Morsy ZS, Helmy H, Ramzy RM, El Setouhy M, Weil GJ (2007). A critical appraisal of molecular xenomonitoring as a tool for assessing progress toward elimination of lymphatic filariasis. Am J Trop Med Hyg.

[42] Boyd A, Won KY, McClintock SK, Donovan CV, Laney SJ, Williams SA (2010). A community-based study of factors associated with continuing transmission of lymphatic filariasis in Leogane, Haiti. PLoS Negl Trop Dis.

[43] Simonsen PE, Derua YA, Kisinza WN, Magesa SM, Malecela MN (2013). Lymphatic filariasis control in Tanzania: effect of six rounds of mass drug administration with ivermectin and albendazole on infection and transmission. BMC Infect Dis.

[44] Nwabufo S, Okiwelu AN (2012). Breeding sites of *Culex quinquefasciatus* (Say) during the rainy season in rural lowland rainforest, Rivers State, Nigeria. Public Health Res.

[45] Emidi B, Kisinza WN (2017). Seasonal variation of *Culex quinquefasciatus* densities emerged from pit-latrines in rural settings, Muheza, Tanzania. SM J Public Heal Epidemiol.

[46] Bockarie MJ, Pedersen EM, White GB, Michael E (2009). Role of vector control in the Global Program to eliminate lymphatic filariasis. Annu Rev Entomol.

[47] Dossou-yovo J, Christian JM, Rivière F (1995). Urbanization and establishment of *Culex quinquefasciatu*s in a West African rural area. Acta Trop.

[48] Mekuriaw W, Balkew M, Messenger LA, Yewhalaw D, Woyessa A, Massebo F (2019). The effect of ivermectin ® on fertility, fecundity and mortality of *Anopheles arabiensis* fed on treated men in Ethiopia. Malar J.

[49] Sampaio VS, Beltrán TP, Kobylinski KC, Melo GC, Lima JBP, Silva SGM (2016). Filling gaps on ivermectin knowledge: effects on the survival and reproduction of *Anopheles aquasalis*, a Latin American malaria vector. Malar J.

[50] Kobylinski KC, Deus KM, Butters MP, Hongyu T, Gray M, da Silva IM (2010). The effect of oral anthelmintics on the survivorship and re-feeding frequency of anthropophilic mosquito disease vectors. Acta Trop.

[51] Sylla M, Gray M, Chapman PL, Sarr MD, Rasgon JL (2010). Mass drug administration of ivermectin in south-eastern Senegal reduces the survivorship of wild-caught, blood fed malaria vectors. Malar J.

[52] Fritz ML, Siegert PY, Walker ED, Bayoh MN, Vulule JR, Miller JR (2009). Toxicity of bloodmeals from ivermectin-treated cattle to* Anopheles gambiae* s.l. Ann Trop Med Parasitol..

[53] Derua YA, Kisinza WN, Simonsen PE (2015). Differential effect of human ivermectin treatment on blood feeding *Anopheles gambiae* and *Culex quinquefasciatus*. Parasit Vectors.

[54] Lyimo IN, Kessy ST, Mbina KF, Daraja AA, Mnyone LL (2017). Ivermectin-treated cattle reduces blood digestion, egg production and survival of a free-living population of *Anopheles arabiensis* under semi-field condition in south-eastern Tanzania. Malar J.

[55] Chavasse DC, Lines JD, Ichimori K, Majala AR, Minjas JN, Marijani J (1995). Mosquito control in Dar es Salaam. II. Impact of expanded polystyrene beads and pyriproxyfen treatment of breeding sites on *Culex quinquefasciatus* densities. Med Vet Entomol..

[56] Mutuku FM, King CH, Mungai P, Mbogo C, Mwangangi J, Muchiri EM (2011). Impact of insecticide-treated bed nets on malaria transmission indices on the south coast of Kenya. Malar J.

[57] Russell TL, Lwetoijera DW, Maliti D, Chipwaza B, Kihonda J, Charlwood JD (2010). Impact of promoting longer-lasting insecticide treatment of bed nets upon malaria transmission in a rural Tanzanian setting with pre-existing high coverage of untreated nets. Malar J.

[58] Clarke SE, Bøgh C, Brown RC, Pinder M, Walraven GEL, Lindsay SW (2001). Do untreated bednets protect against malaria?. Trans R Soc Trop Med Hyg.

[59] Kobylinski KC, Sylla M, Chapman PL, Sarr MD, Foy BD (2011). Ivermectin mass drug administration to humans disrupts malaria parasite transmission in Senegalese villages. Am J Trop Med Hyg.

[60] Chandre F, Hougard JM. Systemic action of ivermectin on *Culex quinquefasciatus* and *Simulium squamosum*. Bull Soc Pathol Exot. 1999;92. 10214528

[61] Yadouléton A, Badirou K, Agbanrin R, Jöst H, Attolou R, Srinivasan R (2015). Insecticide resistance status in *Culex quinquefasciatus* in Benin. Parasit Vectors.

[62] Jones CM, MacHin C, Mohammed K, Majambere S, Ali AS, Khatib BO (2012). Insecticide resistance in *Culex quinquefasciatus* from Zanzibar: implications for vector control programmes. Parasit Vectors.

[63] Lehmann T, Dao A, Yaro AS, Diallo M, Timbiné S, Huestis DL, Adamou A, Kassogué Y (2014). Seasonal variation in spatial distributions of *Anopheles gambiae* in a Sahelian Village: evidence for aestivation. J Med Entomol.

