# Supplementary material for: Lymphatic filariasis, infection status in Culex quinquefasciatus and Anopheles species after six rounds of mass drug administration in Masasi District, Tanzania
Source: Infect Dis Poverty. 2021 Mar 1;10:20. doi: 10.1186/s40249-021-00808-5 (PMC7919328; doi:10.1186/s40249-021-00808-5)
Supplement: Supplementary file 1 — Additional file 1: Checklist on characteristics of the household and the surrounding environment. [file 40249_2021_808_MOESM1_ESM.docx]

**
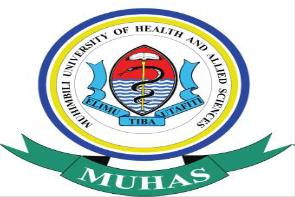
**

**MUHIMBILI UNIVERSITY OF HEALTH AND ALLIED SCIENCES**

**Checklist on characteristics of the study households and the surrounding environment**

**Form No.------------------- Village--------------------------------- Hamlet-------------------**

**Date---------------------------Investigator’s Name------------------**

**Siganture…………………………..**

**PART A: Head of household particular’s.**

**Household ID**-------------------------------

.

**1. Gender** Female Male

**2. Age** ………………….

**3. Marital status**

3.1)Single/not married

3.2) Married

3.3) Divorced

3.4) Widow

**4. Education Level**

4.1) Primary education

4.2) Secondary education

4.3) Advanced/college/university

4.4) Not gone to school

4.5) other (specify) ……………………

**5. Occupation**

5.1) student

5.2) farming/peasant

5.3) cattle grazing

5.4)fishing

5.5) private/public employee

**6) Is there any method you use to protect yourself/your family against mosquitoes?**

6.1) Yes

6.2) No

If your answer is YES answer question number 7

**7. What method do you use to protect yourself/family against mosquitoes?**

7.1) Bednet (s)

7.2) IRS

7.3)Topical repellants

7.4) Mosquito coils

7.5) Local methods (specify)………………………….

**SECTION B: Checklist for household characteristics and the surrounding environment**

**Household ID---------------------------**

**8. House type**

8.1) Mud house

8.2) Brick and grass roof

8.3) Bricks and iron roof

**9. Window**

9.1) Screened

9.2) Not screened

**10. Latrine type**

10.1) Pit latrine

10.2) Modern latrine

**11. Presence of stagnant water**

11.1) Yes

11.2) No

**12. Presence of tall grasses around the household**

12.1) Yes

12.2)No

**13. Presence of bushes around the household**

13.1) Yes

13.2) No

**14. Presence of open waste water chamber**

14.1) Yes

14.2) No

**15. Presence of unused open well around the household**

14.1) Yes

14.2) No
