# Supplementary material for: Lymphatic filariasis, infection status in Culex quinquefasciatus and Anopheles species after six rounds of mass drug administration in Masasi District, Tanzania
Source: Infect Dis Poverty. 2021 Mar 1;10:20. doi: 10.1186/s40249-021-00808-5 (PMC7919328; doi:10.1186/s40249-021-00808-5)
Supplement: Supplementary file 2 — Additional file 2: Questionnaires for community lymphatic filariasis screening in Masasi District council. [file 40249_2021_808_MOESM2_ESM.docx]

**
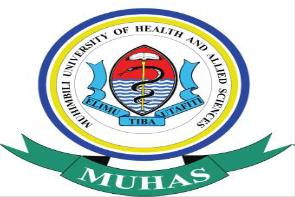
**

**MUHIMBILI UNIVESRSITY OF HEALTH AND ALLIED SCIENCES (MUHAS)**

**QUESTIONNAIRES FOR COMMUNITY LF SCREENING IN MASASI DISTRICT COUNCIL**

**Investigator’s signature …………………….. Date………….** **… …….**

**Identification**

**ID No** ……………………………………

**Village………………… Hamlet………………..**

**A. Social Demographic characteristics**

1. **Gender**: 1.Female 2. Male
2. **Age**…………………….

3**) Marital status**

3.1) Single/ not married

3.2) married

3.3) divorced

3.4) Widow

3.5) Separated

**Page 1 of 7**

**4) Education level**

4.1) Primary

4.2) Secondary

4.3) College/University

4.4) Not gone to school

4.5) other (specify)………………….

1. **Occupation**

5.1)) Student

5.2) farmer/peasant

5.3) cattle grazing

5.4) fishing

5.5) business

5.6) Public/private employee

5.7) Domestic work/housewife

5.8) No occupation

5.9) other (specify) -------------------------------

1. **For how long have you been in this village?**

6.1) 1 to 6months

6.2) 6months to 1year

6.3)1 to 5years

6.4) More than 5 years

6.5) other (specify) ----------------------------------------------

**Page 2 of 7**

**B.Knowledge on LF disease, transmission and prevention**

**7) Have you ever heard of Lymphatic filariasis?**

7.1) Yes

7.2) No

**8) If yes, where did you get the information from?**

8.1) TV

8.2) Radio

8.3) Health workers

8.4) Drug distributor

8.5) other (specify) -------------------------

**9) In your opinion, do you consider Lymphatic filariasis as a health problem in your village?**

9.1) yes

9.2) no (**go to question number 11**)

**10) If yes, why do you think it is a problem?**

10.1) causes frequent fever

10.2) make the diseased weak

10.3) Elephantiasis is shameful

10.4) Hydrocele is shameful

10.5) other (specify) ----------------------------------

**11) If no, why do you think it is not a problem?**

- 1. People can live with it for many years
  2. only a few people have it
  3. other ( specify) -----------------------------------

**Page 3 of 7**

**12) Do you know how a person can get lymphatic filariasis?**

12.1) Yes

12.2) No (**go to question number 14)**

**13) If yes, can you tell me how a person can get lymphatic filariasis?**

13.1) through mosquito bite

13.2) through body contact

13.3) inherited

13.4) Bacteria/virus infection

13.5) Be-witched

13.6) I don’t know

**14) Can LF be prevented?**

14.1) yes

14.2) No (**go to question number 16)**

14.3) I don’t know

**15) If yes, how can LF be prevented?**

15.1) Sleeping under bednets

- 1. Swallowing the drugs which are being distributed by the ministry of health
  2. Use of mosquito repellants
  3. Removing mosquito breeding sites
  4. Avoid contacts with infected person
  5. Using Traditional medicines
  6. I don’t know

**Page 4 of 7**

**16) If no, what are the reasons?**

- 1. Inherited
  2. Because it is bewitchment
  3. Peoples behaviors not easy to control
  4. Other( specify)-------------------------

**C. Particpation in MDA**

**17**) **When did you participate in MDA for Lymphatic filariasis?**

- 1. 2019
  2. 2018
  3. 2017

17.4) other previous years---------

- 1. ) Never ( **go to question number 18)**

**18)** **If you never participated, what was the reason?**

- 1. I was not informed
  2. Was not around during distribution
  3. Fear of side effects
  4. Drug causes infertility
  5. I was not permitted by my elders/parents
  6. I don’t see the importance of it
  7. Personal reasons
  8. I don’t have a reason

**19) If you received the drugs what did you do after receiving them?**

- 1. I swallowed the drugs
  2. I put them somewhere and swallowed later
  3. I didn’t swallow at all
  4. other ( specify) ----------------------------

**Page 5 of 7**

**20) Can you tell me why you didn’t swallow the drugs?**

20.1) I was not given the tablets

- 1. I do not have the disease
  2. I was not there
  3. I don’t like the tablets
  4. I am taking other tablets
  5. Fear of side effects
  6. I don’t think the tablets are effective
  7. I don’t see the importance of it
  8. I was not informed about the reasons for taking the tablets
  9. I have taken alcohol
  10. Was not permitted by elders/parents
  11. Personal reason
  12. I don’t have a reason

**D. Assessment of Bednet Ownership**

**21. Do you have bednet(s) in your household?**

- 1. Yes
  2. No **( go to question number 24)**

**22) If yes, are the bednet(s) treated?**

22.1) yes

- 1. no

**23)** **How many bednets do you have in your house?**

23.1) one

23.2) more than one

23.3) other (specify) --------------------------------------------

**Page 6 of 8**

**24) If no, how do you protect yourself from mosquito bites?**

- 1. Mosquito repellants
  2. Mosquito coils
  3. IRS/insecticide sprays
  4. Other (specify)----------------------------

**25)** **Who uses bed nets in your house?**

25.1) only children below the age of five

25.2) only school children

25.3) elders only

25.4) All household members

25.5) Pregnant women

25.6) other (specify) -------------------------

**26) Did you sleep under ITNs last night?**

26.1) yes

26.2) no

**E. Disease status**

**27) Do you have any symptoms of lymphodema or hydrocele?**

27.1) yes

27.2) no

**28) Have you ever experienced any of the following signs and symptoms?**

- 1. Fevers and limb swelling
  2. Swollen legs
  3. Swollen arms
  4. Swollen scrotum
  5. other ( specify)-----------------------

Page **7** of **8**
